# Screening of Alfalfa Varieties Resistant to *Phytophthora cactorum* and Related Resistance Mechanism

**DOI:** 10.3390/plants12040702

**Published:** 2023-02-05

**Authors:** Menghuan Tao, Yao Zhao, Tianxue Hu, Quan Zhang, Hui Feng, Yiwen Lu, Zhenfei Guo, Bo Yang

**Affiliations:** 1 College of Grassland Science, Nanjing Agricultural University, Nanjing 210095, China; 2 College of Plant Protection, Nanjing Agricultural University, Nanjing 210095, China; 3 Tobacco Research Institute, Chinese Academy of Agricultural Sciences, Qingdao 266101, China

**Keywords:** alfalfa, root rot, resistance, *Phytophthora cactorum*

## Abstract

Alfalfa is one of the most important legume forages in the world. Root rot caused by soil-borne pathogens severely restricts the production of alfalfa. The knowledge of the interaction between alfalfa and root rot-pathogens is still lacking in China. *Phytophthora cactorum* was isolated from symptomatic seedlings of an alfalfa field in Nanjing with high levels of damping-off. We observed the different infection stages of *P. cactorum* on alfalfa, and found that the purified *P. cactorum* strain was aggressive in causing alfalfa seed and root rot. The infecting hyphae penetrated the epidermal cells and wrapped around the alfalfa roots within 48 h. By evaluating the resistance of 37 alfalfa cultivars from different countries to *P. cactorum*, we found Weston is a resistant variety, while Longdong is a susceptible variety. We further compared the activities of various enzymes in the plant antioxidant enzyme system between Weston and Longdong during *P. cactorum* infection, as well as gene expression associated with plant hormone biosynthesis and response pathways. The results showed that the disease-resistant variety Weston has stronger antioxidant enzyme activity and high levels of SA-responsive PR genes, when compared to the susceptible variety Longdong. These findings highlighted the process of interaction between *P. cactorum* and alfalfa, as well as the mechanism of alfalfa resistance to *P. cactorum*, which provides an important foundation for breeding resistant alfalfa varieties, as well as managing *Phytophthora*-caused alfalfa root rot.

## 1. Introduction

Alfalfa (*Medicago sativa*) is the most important perennial forage legume worldwide, with high yields, wide adaptation, and high nutrient quality. Therefore, it is regarded as “the queen of forages” [1]. Rapid and uniform seedling emergence is critical for obtaining a productive stand of alfalfa. However, diseases are significant threats to alfalfa and cause substantial yield losses [2,3]. Many alfalfa seeds are sown in cold, wet soil, which promotes the survival and infection of pathogens and easily leads to seed deterioration and seedling root rot [4]. Many soil-borne pathogens including *Pythium* spp., *Phytophthora* spp., *Fusarium* spp. and *Rhizoctonia solani* can attack alfalfa seeds and seedlings, cause seed rot and root rot of alfalfa [4,5,6]. Previous research on alfalfa diseases focused on fungal pathogens such as *Fusarium* and *Rhizoctonia* due to the limitations of isolation and culture conditions. However, oomycete pathogens such as *Pythium* and *Phytophthora*, which have been found in other leguminous plants such as soybean [4,7], are much more destructive to alfalfa seeds and seedlings.

The hemi-biotrophic oomycete pathogen *P. cactorum* is a destructive and widespread pathogen with a broad host range [8]. It is often responsible for plant root, crown, and collar rots, as well as foliar and fruit infections on herbaceous and woody species, and causes devastating losses to many economically important crops, such as tomato, alfalfa, and strawberry [9,10]. In China, alfalfa root rot caused by *Phytophthora* infection was reported for the first time in Gansu Province in 2018 [10]. The disease’s primary symptoms were red or dark brown lesions on the plants’ main roots, reduced or incomplete growth of lateral roots, and wilting and withering of the overground parts of severely infected plants [10]. Statistics revealed that the prevalence of alfalfa root rot in the afflicted plots was 40–50%, severely reducing alfalfa quality and yield [10]. *P. cactorum* is a homothallic pathogen and produces oospores in the infected plant tissue. These oospores can survive in the soil for many years, even in the absence of a host plant, which acts as an important source of infection in field production systems [11]. Once the environmental circumstances are appropriate, the spread and pathogenicity of *P. cactorum* are fast, and the effectiveness of regular chemical pesticides is limited.

Plants have evolved two layers of the innate immune system to defend against pathogens. The first layer is pattern-triggered immunity (PTI) mediated by pattern recognition receptors that recognize pathogen-associated molecular patterns (PAMPs) or plant-derived damage-associated molecular patterns. The second layer is effector-triggered immunity (ETI) based on the recognition of pathogen effectors by plants nucleotide-binding domain and leucine-rich repeat receptors (NLRs) [12,13]. Pathogen infections trigger various physiological and biochemical responses in plant tissue, such as stimulation of various defense and pathogenesis-related genes expression, upregulation of antioxidant defense enzymes and phytohormones, production of antimicrobial compounds like phytoalexins [14,15]. Superoxide dismutase (SOD), peroxidase (POD), polyphenol oxidase (Ppo), and phenylalanine ammonia-lyase (PAL) are important oxidative enzymes, found in most plant species, that are responsible for scavenging ROS and formation of lignin or other oxidative phenols [16,17]. In addition, phytohormones such as salicylic acid (SA), jasmonic acid (JA), and ethylene (ET), are the classical immune signaling molecules in response to pathogen infection [18,19]. SA is responsible for defense against biotrophic and hemibiotrophic pathogens, like *Pseudomonas syringae* and oomycete *Hyaloperonospora arabidopsidis* [20,21]. On the other hand, JA and ET signaling are reported to be important for immunity against necrotrophic pathogens like *Botrytis cinerea* [22] or *Alternaria brassicicola* [23].

Breeding disease-resistant cultivars is the most economic and effective approach for green management of plant resistance to *P. cactorum*, and also an important strategy to secure the sustained growth of green agriculture [9,11]. The interaction between *P. cactorum* and host plants is consistent with the “gene to gene” concept, which states that for *Phytophthora* with certain avirulence genes, host plants with corresponding resistance genes can effectively inhibit the infection [12,13]. The main mode of infection of *P. cactorum*, like most *Phytophthora*, is by mobile, mononuclear, double-flagellated zoospores. They swim towards the roots of plants in water after being released from the sporangia. Once attached to the plant, zoospore flagella fall off, germinate, and penetrate host cells. However, there is a limited reference on the infection of *P. cactorum* on alfalfa.

To investigate the interaction between *P. cactorum* and alfalfa, and to identify the resistance of different alfalfa varieties to *Phytophthora*, in this study, (i) we isolated and identified *P. cactorum* strain Pca-NJ-1 from infected alfalfa plants in the field, confirming the strong pathogenicity of *P. cactorum* on alfalfa; (ii) we observed the different infection stages of *P. cactorum* on alfalfa and evaluated the resistance of 37 alfalfa cultivars from different countries to *P. cactorum* in the laboratory; (iii) we chose a disease resistant and a susceptible cultivar to detect the activities of various enzymes in the plant antioxidant enzyme system during *P. cactorum* infection, as well as gene expression associated with plant hormone biosynthesis and response pathways. These findings highlighted the process of interaction between *P. cactorum* and alfalfa, as well as the mechanism of alfalfa resistance to *P. cactorum*, which provides an important foundation for breeding resistant alfalfa varieties and selecting varieties for planting in different regions, as well as managing and control *Phytophthora*-caused alfalfa root rot.

## 2. Results

### 2.1. Isolation and Identification of Alfalfa Root Rot Pathogen P. cactorum

**Figure 1:**
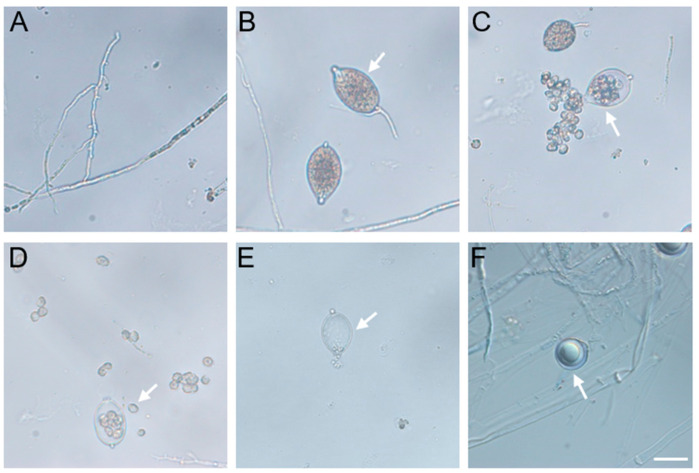
Morphological characteristics of *Phytophthora cactorum*. (**A**) Morphology of hyphae. (**B**) Pyriform sporangia. (**C**) Sporangia that are releasing zoospores. (**D**) Zoospores. (**E**) Empty sporangium. (**F**) Oospore. Arrows indicate morphological characteristics. Scale bars: 40 μm.

Using the V8 medium, we identified numerous pathogens from typical diseased alfalfa samples from Nanjing Agricultural University’s Baima research base. One of these isolates was identified as *P. cactorum* and named Pca-NJ-1 after amplification and sequencing of its *ITS* and *COX1* genes. After single spore isolation, morphological characteristics of *P. cactorum* were observed on the V8 plate. *P. cactorum* colonies on the V8 medium were uniform, with clear and obvious margins and fewer aerial mycelium (Figure S1). Hyphae are coenocytic, usually less than 6 μm wide, and often irregularly shaped with randomly placed, slight swellings and loose coralloid growth (Figure 1A). Microscopic observation revealed that the mycelium was branched (Figure 1A). *P. cactorum* produces caducous, usually terminal, ovoid, ellipsoid, or pear-shaped sporangia, each on a short pedicel (Figure 1B). Sporangia have prominent papillae and are typically 35 μm long (+/− 5 μm) and 26 μm (+/− 4 μm) wide (Figure 1B). After the sporangia matured, the zoospores were released from one side, which was ovoid and about 10 μm (+/− 2 μm) in size (Figure 1C,D). At releasing moment, the zoospores could be clearly seen to be retarded in the vesicular membrane structure, and eventually, the membrane structure ruptured under the compression of the zoospores, which then spread out in all directions (Figure 1C,D). After release, zoospores swim away with the water, leaving empty sporangia (Figure 1E). Sexual reproduction in *P. cactorum* is uniformly homothallic with paragynous antheridia. The diameters of oospores are typically 30 μm (+/− 5 μm) (Figure 1F).

**Figure 2:**
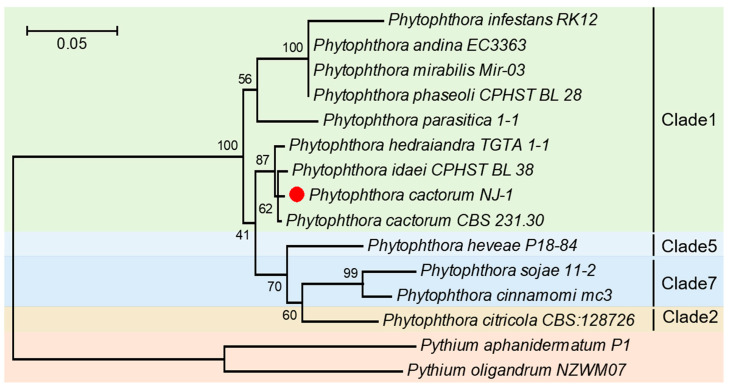
Molecular phylogenetic analysis by Maximum Likelihood method. The evolutionary history was inferred by using the Maximum Likelihood method based on the Tamura-Nei model. The tree with the highest log likelihood (−3414.8106) is shown. The percentage of trees in which the associated taxa clustered together is shown next to the branches. Initial tree(s) for the heuristic search were obtained automatically by applying Neighbor-Join and BioNJ algorithms to a matrix of pairwise distances estimated using the Maximum Composite Likelihood (MCL) approach, and then selecting the topology with superior log likelihood value. The tree is drawn to scale, with branch lengths measured in the number of substitutions per site. The analysis involved 15 nucleotide sequences. Codon positions included were 1st + 2nd + 3rd + Noncoding. All positions with less than 95% site coverage were eliminated. That is, fewer than 5% alignment gaps, missing data, and ambiguous bases were allowed at any position.

The ITS sequence of the isolate Pca-NJ-1 was used to construct a phylogeny tree with the ITS sequences of other closely related oomycete species. These included *P. cactorum*, *P. infestans*, *P. phaseoli*, *P. idaei*, *P. andina*, *P. parasitica*, *P. hedraiandra*, *P. mirabilis* belonging to Clade1, *P. citricola* of Clade2, *P. heveae* of Clade5, *P. cinnamomi* and *P. sojae* of Clade7. The results showed that it was most closely related to the standard strain *P. cactorum* CBS231.30 and was clustered into one branch, which was further determined as *P. cactorum* (Figure 2).

### 2.2. Pathogenicity Test Confirmed That P. cactorum Caused Severe Seed and Root Rot of Alfalfa

**Figure 3:**
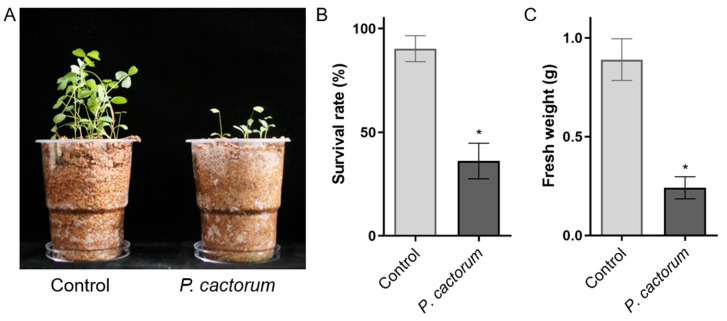
Symptoms, survival rates, and fresh weight of alfalfa inoculated with or without *Phytophthora cactorum*. (**A**) Growth phenotypes of alfalfa inoculated with *P. cactorum* or not in pot. (**B**) Survival rates and (**C**) fresh weight of alfalfa inoculated with *P. cactorum* or not. Data are the means of six independent experiments and error bars represent SD. Asterisks at the top of the bars indicate statistical significance (T-test, *p* < 0.05).

After identifying the isolated strain Pca-NJ-1 as *P. cactorum*, we tested its pathogenicity on alfalfa. The mycelium growing on V8 medium was chopped and mixed with sterile vermiculite before sowing alfalfa seeds (cultivar Aurora) that had been soaked in sterile water for 12 h. The disease phenotype was observed after 14 days. The results showed that the survival rate of alfalfa was significantly reduced after inoculation with Pca-NJ-1 compared with the non-inoculated control, with nearly 60% of the seeds failing to emerge, and the surviving plants were significantly shorter, had shorter root systems, and had brown spots on the main roots (Figure 3A,B). The biomass of alfalfa was found to be significantly reduced after inoculation with Pca-NJ-1 (Figure 3C), which indicated that *P. cactorum* was capable of causing severe seed and root rots on alfalfa.

### 2.3. Staining Observation on the Infection of Alfalfa by P. cactorum

**Figure 4:**
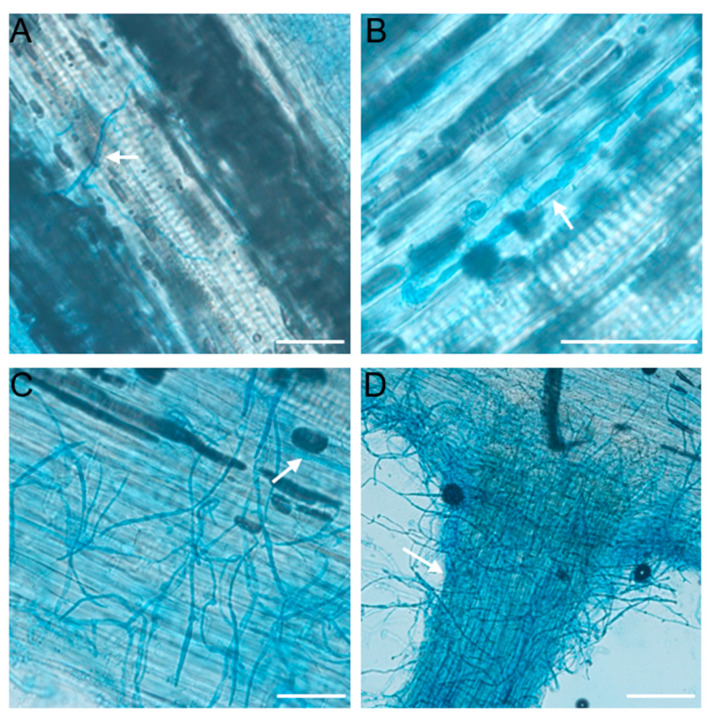
Photo of alfalfa root staining after *Phytophthora cactorum* infection. (**A**,**B**) Invasive hypha grows intercellularly in epidermal cells. Different root tissues were stained with the lactophenol-trypan blue for 30 s before microscope observation. Infectious growth was observed at 24 hpi. (**C**) Invasive hypha grows in/outside of alfalfa cells. Infectious growth was observed at 36 hpi. (**D**) Typical infection sites of alfalfa roots inoculated with *P. cactorum* strain, showing stained mycelium wrapped around alfalfa roots and proliferation within tissue. Infectious growth was observed at 48 hpi. Scale bars = 100 μm. The arrow indicates typical infection hypha.

In order to further investigate the infection of alfalfa by *P. cactorum*, we first induced the production of zoospore of *P. cactorum* and inoculated the alfalfa roots by soaking them in zoospore suspension, and then stained the infested tissues using lactophenol-trypan blue to observe the infection. At 24 h post-infection (hpi), *P. cactorum* mycelium expanded on the surface of alfalfa roots was observed (Figure 4A). The infested hyphae formed thicker hyphae within the cells and irregular tumor-like hyphae appear (Figure 4B). At 36 hpi, the infected hyphae penetrated the alfalfa cells, and there were a large number of hyphae inside and on the surface of the alfalfa cells (Figure 4C). At 48 hpi, the infected hyphae had penetrated the epidermal cells and expanded outward, and a large number of hyphae had been wrapped around the alfalfa roots, and cell death began to occur in some cells (Figure 4D).

### 2.4. Evaluation of the Resistance Level of Different Alfalfa Varieties to P. cactorum

**Figure 5:**
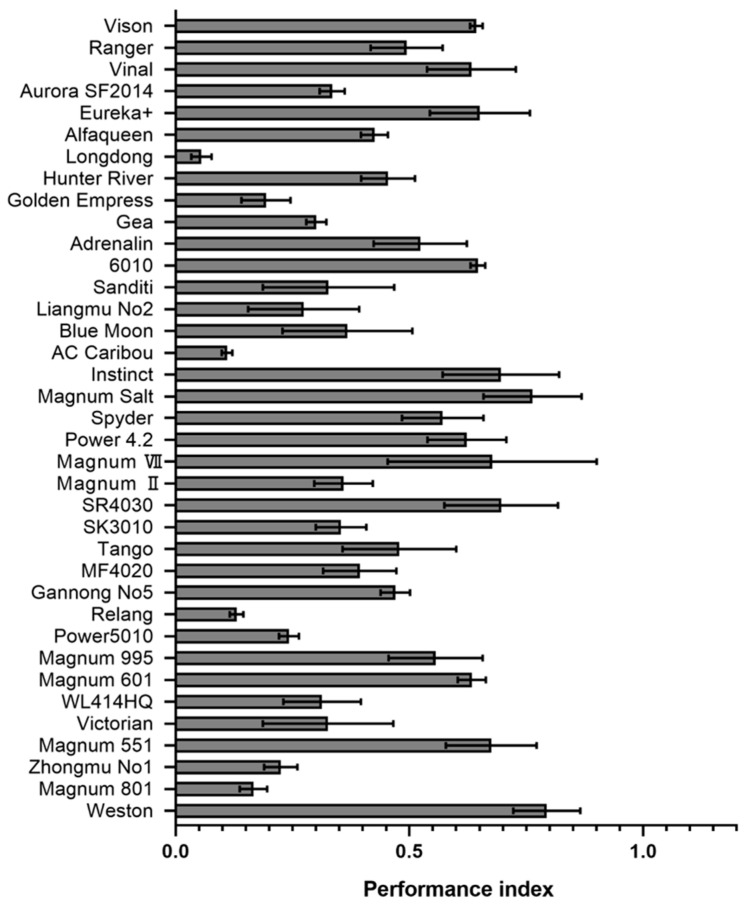
Performance of different alfalfa varieties after *Phytophthora cactorum* infection. Performance index (Fresh weights of 2-week-old plants normalized by germination rate) of alfalfa plants inoculated with *P. cactorum*. At least three independent biological replicates were performed for each treatment. Plant performance index (PPI) (PPI = Relative fresh weights (RFW) × Relative survival rate (RSR).

After confirming the pathogenicity of *P. cactorum* to alfalfa, we further evaluated the resistance to *P. cactorum* of 37 uncoated alfalfa seeds from domestic and foreign sources collected and preserved in our lab. Using mycelial agar plug inoculation experiments, we tested the incidence of these different varieties 14 days after planting. To exclude the effects caused by differences in the germination rate of the seeds themselves, we set up separate inoculated and uninoculated groups and calculated the relative survival rates and fresh weight (Table S2). The results showed that the resistance of 37 different varieties of alfalfa to *P. cactorum* varied, and the relative survival rates ranged from 20% to 96% (Table S2). We identified that seed inoculation with *P. cactorum* influenced both relative survival rate (RSR = the survival rate of the inoculation group/the survival rate of the control group, Table S2) and relative fresh weight (RFW = the fresh weight of the inoculation group/the fresh weight of the control group, Table S2) of two-week-old plants, and therefore calculated a plant performance index (PPI = RFW × RSR) (Table S2). PPI data show that the four most susceptible species to *P. cactorum* are Longdong (PPI = 0.06), AC Caribou (PPI = 0.11), Relang (PPI = 0.13), Magnum 801 (PPI = 0.17). The four highest disease-resistant varieties were Weston (PPI = 0.79), Magnum Salt (PPI = 0.76), Instinct, and SR4030 (PPI = 0.70) (Figure 5).

### 2.5. Microscopic Observation of Resistant or Susceptible Alfalfa Varieties Infected by P. cactorum at Different Time Points

**Figure 6:**
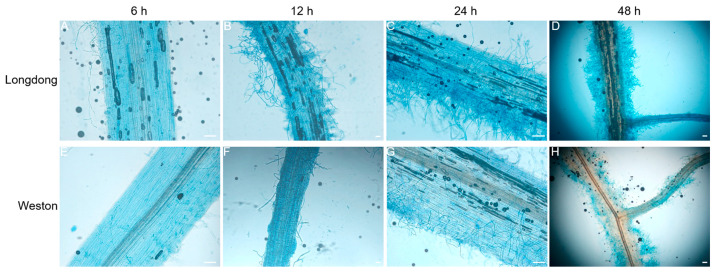
Microscopic observation of resistant or susceptible alfalfa varieties infected by *Phytophthora cactorum* at different time points. (**A**–**D**) Typical infection sites of Longdong roots inoculated with the *P. cactorum* strain, showing greater mycelium proliferation and tissue invasion within tissue. (**E**–**H**) The infection sites of Weston infected with *P. cactorum*, showing less mycelial infection. Different root tissues were stained with the lactophenol-trypan blue for 30 s before microscope observation. Infectious growth was observed from 6 hpi to 24 hpi. Scale bars = 200 μm.

From the 37 alfalfa varieties mentioned above, we selected the most resistant variety Weston and the most susceptible variety Longdong, and further inoculated the alfalfa through the root soaking method of zoospores of *P. cactorum*, and took samples at different time points after inoculation to observe the infection. At 6 h post infection (hpi), the infected hyphae had sprouted on the root surface of the susceptible variety Longdong and invaded into the surface cells (Figure 6A), while almost no hyphae were present on the root surface of the resistant variety Weston (Figure 6E). At 12 hpi, Longdong roots were already heavily entangled with mycelium (Figure 6B), while the amount of mycelium around Weston’s roots was still low (Figure 6F). With the extension of the infection time, by 24 hpi, both varieties had a mycelial infestation in the roots, but compared to Weston, Longdong roots had significantly more mycelial colonization, and cell death occurred in the tissues (Figure 6C,G). At 48 hpi, Longdong roots were already surrounded by mycelium and the tissues appeared brownish and necrotic (Figure 6D), while a certain amount of mycelium was also present in Weston roots, but the amount was significantly lower than that in Longdong roots, and Weston roots still looked relatively healthy (Figure 6H).

### 2.6. Antioxidant Enzyme Activities of Resistant or Susceptible Alfalfa Varieties Infected by P. cactorum at Different Time Points

**Figure 7:**
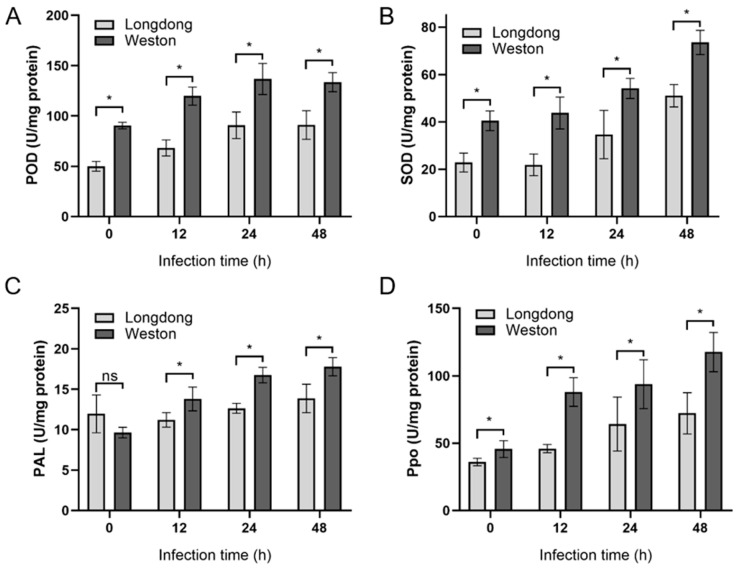
Antioxidant enzyme activities of resistant or susceptible alfalfa varieties infected by *Phytophthora cactorum* at different time points. (**A**) Peroxidase (POD), (**B**) Superoxide dismutase (SOD), (**C**) Phenylalanine ammonia-lyase (PAL), (**D**) Polyphenol oxidase (Ppo) were measured at 0, 12, 24 or 48 hpi. Data are the means of six independent experiments and error bars represent SD. Asterisks at the top of the bars indicate statistical significance (T-test, *p* < 0.05).

To investigate the mechanisms of resistance differences between Longdong and Weston, we examined the activities of antioxidant enzyme in plants infected by *P. cactorum* at different time points. The activity of peroxidase (POD), superoxide dismutase (SOD), phenylalanine ammonia-lyase (PAL) and Polyphenol oxidase (Ppo) were detected at 0, 12, 24 and 48 hpi. The results showed that activities of POD, SOD, PAL and Ppo were increased after *P. cactorum* infection, and the activities of different enzymes in both resistant and susceptible varieties were highest after 48 hpi, except for the Weston sample where POD activity was almost the same at 24 h and 48 hpi (Figure 7A–D). Moreover, higher levels of POD, SOD, PAL and Ppo were detected in roots in Weston than that in Longdong (Figure 7A–D), suggesting that the disease-resistant variety has stronger antioxidant enzyme activity in its roots.

### 2.7. Relative Expression of Phytohormone-Related Genes in Resistant or Susceptible Alfalfa Varieties Infected by P. cactorum at Different Time Points

**Figure 8:**
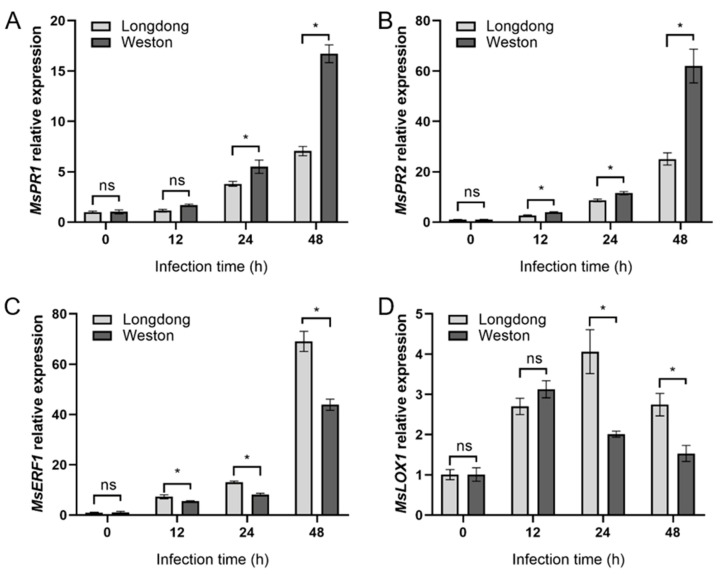
Relative expression of plant hormone-related genes in resistant or susceptible alfalfa varieties infected by *Phytophthora cactorum* at different time points. Expression of (**A**) *MsPR1*, (**B**) *MsPR2*, (**C**) *MsERF1*, (**D**) *MsLOX1* were detected at 0, 12, 24, or 48 hpi. Data are the means of six independent experiments and error bars represent SD. Asterisks at the top of the bars indicate statistical significance (T-test, *p* < 0.05).

To further investigate the mechanisms underlying the differences in disease resistance between the disease-resistant variety Weston and the susceptible variety Longdong, we further determined the expression of phytohormone response and synthesis-related genes, such as PR1 and PR2 associated with salicylic acid response, LOX1 associated with jasmonic acid, and ERF1 associated with ethylene response, in plant root samples after infection at different time points. The results showed that, in terms of the overall trend, the expression of *MsPR1*, *MsPR2*, *MsLOX1,* and *MsERF1* showed up-regulated expression as the time of pathogen infection increased in both disease-resistant and susceptible varieties (Figure 8A–D). For the salicylic acid-responsive gene *MsPR1*, its expression was not significantly different between the disease-resistant variety Weston and the disease susceptible variety Longdong at 0 hpi and 12 hpi, while when it comes to 24 hpi and 48 hpi, the expression level of *MsPR1* in Weston samples was much higher than that in Longdong (Figure 8A). For *PR2*, the expression of *MsPR2* in Weston samples was already significantly higher than that in Longdong after 12 hpi. At 0 hpi, there was no significant difference in *MsPR2* expression in Weston and Longdong (Figure 8B). In contrast, the expression of *MsERF1* in Weston was significantly lower than that in Longdong after 12 hpi (Figure 8C), and the expression of *MsLOX1* was also significantly lower than that of Longdong after 24 hpi (Figure 8D).

## 3. Discussion

Alfalfa is the most important legume forage. In recent years in China, as people’s need for meat, eggs, and milk has increased, the demand for alfalfa has increased greatly. China imported 1.78 million tons of alfalfa in 2021 [24,25]. Improving the productivity of alfalfa will enhance national food security. Alfalfa root rot is a crucial factor limiting alfalfa yield. The pathogens that cause root rot are diverse, with the majority of them being pathogenic oomycetes like *Phytophthora* and *Pythium*, as well as pathogenic fungi like *Rhizoctonia solani* and *Fusarium oxysporum* [3,6,26]. However, due to a lack of attention given to alfalfa diseases in China or a limitation in isolation methods, the pathogens of alfalfa root rot previously described were primarily limited to pathogenic fungi. V8 media is a unique medium used for the isolation of Oomycete. Furthermore, oomycetes cause seed rot and root rot, which often occur at the seed stage, resulting in no emergence; or seedlings damping off at the early stage, it’s easy to miss the right time to sample the infected tissue. It has been reported that *Pythium* have been associated with severe alfalfa seed rot and root rot outside China [4]. In 2018 in China, Gansu Province reported the first case of the root rot of alfalfa caused by *P. cactorum*, and fields in the severely affected regions reached 50% disease incidence [10]. In Nanjing, we isolated and identified *P. cactorum* strains from the alfalfa field. The root rot of alfalfa caused by *P. cactorum* has been discovered in both northern and southern China, which has aroused our concern. For other *Phytophthora* species, such as *P. sojae* and *P. infestans*, the interaction mechanisms between *Phytophthora* and its host have been well-studied [13]. For example, the RXLR effector of the key *Phytophthora* virulence factor and the disease resistance gene in the corresponding host plants were identified [27,28,29]. Despite the fact that *P. cactorum* has a wide host range, there are currently little research on the interaction between *P. cactorum* and its host, with the majority of them focusing on cash crops such as strawberries [9,11]. It was reported that after *P. cactorum*-infected strawberries and tobacco, the expression of many effectors was up-regulated, which had virulence functions during infection [9]. The establishment of an alfalfa-*Phytophthora* interaction research system is critical for studying *P. cactorum* pathogenesis and alfalfa disease resistance mechanisms. We developed the infection system and revealed the infection progress of *P. cactorum* in this study, and further work will focus on its pathogenic mechanisms.

We tested 37 alfalfa varieties for disease resistance to *P. cactorum* and identified multiple resistant varieties, such as Weston. Further comparison revealed that the antioxidant enzyme system of the resistant variety responds much stronger than that of the susceptible varieties, as reflected by SOD, POD, PAL and Ppo enzyme activities. Although the enzyme activities of resistant and susceptible varieties increased with infection time, the increase in resistant varieties was much greater than that in susceptible varieties. Similar results were also found in the comparison of other plant-resistant and susceptible varieties in response to pathogen infection [30,31]. This indicates that the antioxidant enzyme system plays an important role in plant immunity. Furthermore, the homeostasis and responses of phytohormones play important roles in plant–microbe interactions [32]. We further analyzed the expression of key genes for phytohormone synthesis and signaling, such as the SA-responsive genes *PR1* and *PR2*, the JA synthesis-related gene *LOX1*, and the ethylene-responsive transcription factor *ERF1*. The results showed that the expression up-regulation levels of *PR1* and *PR2* were significantly higher in the disease-resistant variety Weston than in the disease-susceptible variety Longdong, while the expression up-regulation levels of *LOX1* and *ERF1* were significantly lower than in disease-susceptible variety after infected by *P. cactorum*. This result is consistent with the previous research results [20,21]. It was reported that *P. cactorum* is a hemi-biotrophic pathogen [9]. SA plays an important role in promoting plants’ immunity in response to biotrophic and hemibiotrophic pathogens, while JA mediates plants defense against necrotrophic pathogens [20]. Moreover, several studies have shown that the SA signaling pathway predominantly acts antagonistically to JA/ET-mediated signaling [32,33].

*PR1* and *PR2* are commonly used as markers for SA-mediated activation of SAR (systemic acquired resistance) [34]. The antimicrobial functions of the PR proteins have not yet been established. PR2 encodes b-1,3-glucanase [35], while the knowledge of the biochemical function of *PR1* is limited. However, it has been recently reported that the PR1 protein can bind sterols (and this function is responsible for antimicrobial activity) and harbors an embedded defense signaling peptide involved in plant immune signaling [36,37]. The expression levels of PR1 and PR2 that we found were significantly higher in resistant varieties and may be one of the important reasons that contribute to resistance, which could subsequently be used to modify alfalfa resistance to *P. cactorum* based on this result.

## 4. Materials and Methods

### 4.1. Sample Collection

Diseased alfalfa sample (cultivar Aurora) for pathogen isolation is collected from an alfalfa field in the Lishui district, Nanjing, China (Baima teaching and research base, 119°18′07″ E, 31°61′47″ N).

### 4.2. Isolation and Identification

Fresh tissues from symptomatic alfalfa were cut into pieces (3–5 mm) and surface sterilized with 75% ethanol for 10 s, followed by 1% sodium hypochlorite (NaClO) for 120 s, and three rinses with sterile distilled water. Tissue pieces were placed on selective V8 juice agar containing 50 mg/L rifampicin and ampicillin. Plates were sealed and incubated at 25 °C for 48 to 72 h. Colonies were observed in the V8 plate. Take a bit of culture through inoculating needle, incubate at 25 °C for 48 to 72 h. Then purify the isolates by single spore isolation through the serial dilution method. Purified isolates were maintained in a 25 °C incubator. Colony morphology and colors were observed with naked eyes and morphological characteristics of conidia were observed under a microscope. Genome DNA of isolates was extracted for PCR amplification using the CTAB method [38]. ITS and COX1 genes were amplified with corresponding designed primers [39] (Table S1). The amplified fragments were subjected to DNA sequencing and compared with the known sequence homology in GenBank using nucleotide BLAST to determine the pathogen species. The Sequences of related species were selected for comparative phylogenetic analysis.

### 4.3. Pathogenicity Test

*P. cactorum* isolate Pca-NJ-1 was grown in 9 cm Petri dishes containing 15 mL V8 medium and cultured for 5 days at 25 °C. The pathogenicity of Pca-NJ-1 was determined by using the mycelial agar plug inoculation method. Briefly, the medium filled with fresh mycelium was chopped and mixed with vermiculite, and one dish of medium was added to each pot (The upper diameter of the pot is 7 cm and the lower diameter is 5 cm). Alfalfa seeds were soaked in sterilized water for 12 h and then grown in sterilized vermiculite mixed with mycelium pieces and maintained in a greenhouse for 14 days at 25 °C under a 14 h/10 h light/dark cycle. Ten seeds were planted in each pot, and each experiment was repeated six times. Survival rate and fresh weight were recorded. Relative survival rate (RSR) and relative fresh weight (RFW) were calculated, and therefore calculated a plant performance index (PPI = RFW × RSR) [40].

### 4.4. Source of Tested Alfalfa Cultivars

The 37 alfalfa varieties are from different research institutes and companies and were stored in the Grass Biotechnology and Breeding Laboratory of Nanjing Agricultural University.

### 4.5. qRT-PCR Analysis

Samples were collected and ground in liquid nitrogen. Total RNA was extracted using a TRIzol reagent (Thermo Fisher Scientific, Beijing, China) following the manufacturer’s instructions. cDNA synthesis was performed using a HiScript II 1st Strand cDNA Synthesis Kit (Vazyme Biotech Co., Ltd., Nanjing, China) following the manufacturer’s instructions. qRT-PCR was conducted in a 20 µL reaction mix containing 20 ng cDNA, 0.4 µL primers (10 µM) specific for the target or reference gene, 10 µL SYBR Green Premix Ex Taq, 0.4 µL ROX Reference Dye II (50×), and 6.8 µL sterilized deionized water. qRT-PCR was run on the Applied Biosystems 7500 Real-Time PCR System under the following conditions: 95 °C for 30 s, followed by 40 cycles at 95 °C for 5 s and 60 °C for 34 s. Melt curves are routinely run for qPCR experiments to ensure primer specificity, with data typically collected over a temperature range of 60–95 °C in 0.5 °C increments. Mean Ct values were subjected to normalization. There are three biological replicates of each sample. Relative expression was calculated using the normalized (2^−ΔΔCt^) value. The *MsActin* gene was used as the internal control. All gene-specific primers used for qRT-PCR are listed in Table S1.

### 4.6. Microscopic Examination

To monitor the morphological characteristics of *P. cactorum*, strain Pca-NJ-1 was grown on 10% V8 agar medium at 25 °C in the dark. After 7 days, the cultures were examined via microscopy (Olympus). The diameter of sporangia, zoospores, and oospores in three random fields was measured using microscope graticules. To induce the production of zoospores, the *P. cactorum* was analyzed after culturing for 3 days at 25 °C on 10% V8 broth. The plate was rinsed twice with sterile distilled water, flooded with sterile distilled water to cover the mycelium, and then left overnight at 25 °C to release the zoospores.

To assess colonization of alfalfa tissues by *P. cactorum*, roots of two-week-old alfalfa seedlings were soaked in zoospores suspension (containing approximately 10,000 zoospores per mL) for 30 min before being transferred to a sterile Petri dish. Root tissues were collected at different time points after infection and soaked in lactophenol-trypan blue, and then the infected epidermal cells were examined under an inverted microscope. Mycelium stained with the lactophenol-trypan blue (10 mL lactic acid, 10 mL glycerol, 10 g phenol, and 10 mg trypan blue dissolved in 10 mL distilled water) was randomly selected for examination under an inverted microscope (Carl Zeiss, Jena, Germany).

### 4.7. Phylogenetic Analysis of P. cactorum

Phylogenetic trees of the isolated *P. cactorum* strain Pca-NJ-1 with other oomycetes including several *Phytophthora* pathogens and *Pythium* were constructed based on the ITS sequence using the maximum-likelihood (ML) method in MEGA 7.0 software.

### 4.8. Measurements of Plant Antioxidant Enzyme Activities

Two-week old Longdong and Weston seedlings grown in MS medium were collected and were soaked in *P. cactorum* zoospores suspension for 30 min before being transferred to a sterile Petri dish. Root tissues were collected at different time points after infection. Antioxidant enzyme activities were analyzed according to Zhuo et al. [41] and Soheili-Moghaddam et al. [42]. There are three biological replicates of each sample.

### 4.9. Statistical Analysis

All measurements were repeated more than three times. Differences among means of plant lines or treatments were evaluated by Duncan’s test at a 0.05 probability level. All statistical analyses were performed by Statistical Package for the Social Sciences (SPSS 17.0).

## 5. Conclusions

These results further confirmed that *P. cactorum* is the main pathogen of alfalfa root rot in China. *P. cactorum* zoospores were able to colonize alfalfa plants within 12 h and reduce their survival rate and biomass significantly. Importantly, we established the alfalfa- *P. cactorum* inoculation system and revealed the process of interaction between *P. cactorum* and alfalfa, as well as the mechanism of alfalfa resistance to *P. cactorum*, which provides an important foundation for breeding resistant alfalfa varieties and selecting varieties for planting in different regions, as well as to manage and control *Phytophthora*-caused alfalfa root rot.

## Data Availability

The data that support the findings of this study are available from the corresponding author upon reasonable request.

## Supplementary Materials

The following supporting information can be downloaded at: https://www.mdpi.com/article/10.3390/plants12040702/s1, Figure S1: Colony morphology of *P. cactorum* on V8 medium; Table S1: Primers used in this study; Table S2: Performance of different alfalfa varieties after *P. cactorum* infection.
