# Supplementary material for: Screening of Alfalfa Varieties Resistant to Phytophthora cactorum and Related Resistance Mechanism
Source: Plants (Basel). 2023 Feb 5;12(4):702. doi: 10.3390/plants12040702 (PMC9966651; doi:10.3390/plants12040702)
Supplement: Supplementary file 1 [file plants-12-00702-s001.zip › plants-2186205-supplementary.pdf]

Screening of alfalfa varieties resistant to *Phytophthora cactorum* and related resistance mechanism

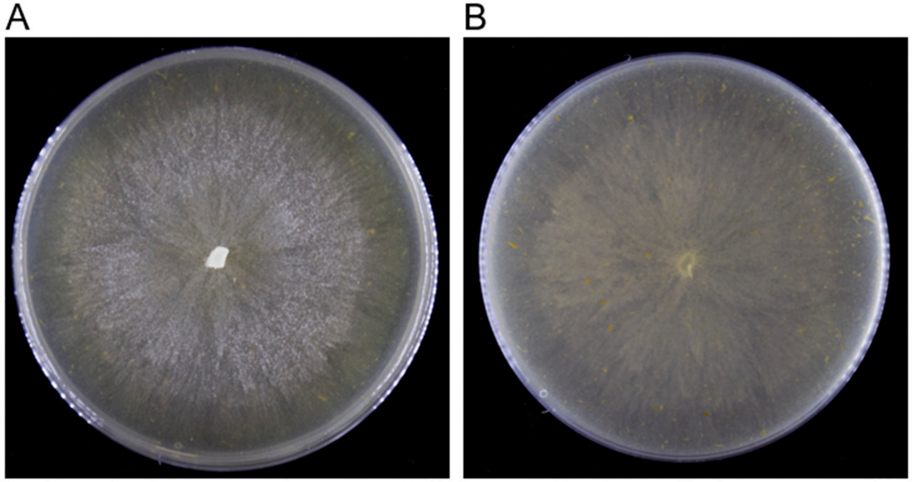

Figure S1. Colony morphology of *P. cactorum* on V8 medium.

(A) The front of the colony. (B) The back of the colony. The newly inoculated colonies were cultured at 25 °C for 5 d and photographed for colony morphology.

Table S1 Primers used in this study

Primer name	Sequence	Usage
ITS1	TCCGTAGGTGAACCTGCGG	For strain identification
ITS4	TCCTCCGCTTATTGATATGC	
CoxI-F	CYTCHGGRTGWCCRAAAAACCAAA	
CoxI-R	TCAWCWMGATGGCTTTTTTCAAC	
MsPR1-1F	AACGAAGCAAGACGTCAGGT	For qRT-PCR
MsPR1-1R	CTCACTGCATCTGTGCCACT	
MsPR2-1F	TGGGACACCAATGAGGTCTG	
MsPR2-1R	TTCCCAATCCATTTTCCCTTCA	
MsERF-1F	GTCGTCCTTGGGGGAAATAC	
MsERF-1R	AAGCTGCAGCAACAGTTTCA	
MsLOX-1F	AGGCCGGAACGTATCTATGC	
MsLOX-1R	AGGTTGGTAGAGTGGGGACA	
MsACTIN-1F	ATTCACGAGACCACCTAC	
MsACTIN-1R	GAGCCACAACCTTAATCTTC	

Table S2 Performance of different alfalfa varieties after *P. cactorum* infection

Number	Variety	Relative Surival Rate (RSR)	Relative Fresh Weight (RFW)	Performance index (PPI)
1	Weston	0.96^{j}	0.83^{lm}	0.79^{p}
2	Magnum 801	0.45^{abc}	0.37^{abcd}	0.17^{abc}
3	Zhongmu No1	$0.69^{bcdefghij}$	0.33^{abc}	0.22^{abcde}
4	Magnum 551	$0.77^{cdefghij}$	0.88^{m}	0.68^{mnop}
5	Victorian	0.59^{bcdefg}	$0.58^{defghijkl}$	$0.32^{abcdefgh}$
6	WL414HQ	0.48^{abcd}	$0.65^{fghijklm}$	$0.31^{abcdefgh}$
7	Magnum 601	$0.77^{cdefghij}$	0.83^{lm}	$0.63^{jklmnop}$
8	Magnum 995	$0.72^{bcdefghi}$	0.77^{klm}	$0.56^{ghijklmnop}$
9	Power5010	0.59^{bcdefg}	0.41^{abcdef}	0.24^{abcdef}
10	Relang	$0.64^{bcdefghi}$	0.21^{a}	0.13^{ab}
11	Gannong No5	$0.63^{bcedfghi}$	0.75^{jklm}	$0.47^{defghijklmn}$
12	MF4020	$0.60^{bcdefgh}$	$0.66^{ghijklm}$	$0.39^{bcdefghijklm}$
13	Tango	$0.68^{bcdefghij}$	0.7^{hijklm}	$0.48^{defghijklmno}$
14	SK3010	$0.70^{bcdefghij}$	$0.50^{cdefghi}$	$0.35^{bcdefghij}$
15	SR4030	0.85^{fghij}	0.82^{klm}	0.70^{nop}
16	Magnum II	$0.80^{defghij}$	0.45^{bcdefg}	$0.36^{bcdefghijk}$
17	Magnum VII	0.87^{fghij}	0.77^{klm}	0.68^{mnop}
18	Power 4.2	0.81^{efghij}	0.76^{klm}	$0.62^{ijklmnop}$
19	Spyder	$0.74^{bcdefghij}$	0.77^{klm}	$0.57^{hijklmnop}$
20	Magnum Salt	0.92^{ij}	0.83^{lm}	0.76^{op}

21	Instinct	0.92^{ij}	0.75^{jklm}	0.70^{nop}
22	AC Caribou	0.44^{ab}	0.25^{ab}	0.11^{ab}
23	Blue Moon	$0.63^{bcdefghi}$	$0.58^{defghijkl}$	$0.37^{bcdefghijkl}$
24	Liangmu No2	0.92^{hij}	0.29^{abc}	$0.27^{abcdefg}$
25	Sanditi	0.56^{bcdef}	$0.57^{defghijk}$	$0.32^{abcdefgh}$
26	6010	0.83^{fghij}	0.79^{klm}	0.64^{klmnop}
27	Adrenalin	0.88^{ghij}	$0.59^{defghijkl}$	$0.52^{fghijklmnop}$
28	Gea	$0.64^{bcdefghi}$	$0.47^{bcdefgh}$	$0.30^{abcdefgh}$
29	Golden Empress	0.50^{abcde}	0.38^{abcde}	0.19^{abcd}
30	Hunter River	$0.73^{bcdefghij}$	$0.63^{fghijklm}$	$0.45^{cdefghijklmn}$
31	Longdong	0.20^{a}	0.29^{abc}	0.06^{a}
32	Alfaqueen	0.83^{fghij}	$0.51^{cdefghij}$	$0.43^{cdefghijklmn}$
33	Eureka+	0.82^{fghij}	0.79^{klm}	0.65^{lmnop}
34	Aurora SF2014	$0.68^{bcdefghij}$	$0.49^{cdefghi}$	$0.33^{abcdefghi}$
35	Vinal	0.85^{fghij}	0.74^{jklm}	$0.63^{jklmnop}$
36	Ranger	$0.80^{defghij}$	$0.62^{efghijkl}$	$0.49^{efghijklmno}$
37	Vison	0.88^{ghij}	0.73^{ijklm}	0.64^{klmnop}

Different letters at the end of the data indicate statistical significance (one-way ANOVA, $P < 0.05$).
